# High Fat Diet Multigenerationally Affects Hippocampal Neural Stem Cell Proliferation via Epigenetic Mechanisms

**DOI:** 10.3390/cells11172661

**Published:** 2022-08-27

**Authors:** Francesca Natale, Matteo Spinelli, Saviana Antonella Barbati, Lucia Leone, Salvatore Fusco, Claudio Grassi

**Affiliations:** 1 Department of Neuroscience, Università Cattolica del Sacro Cuore, 00168 Rome, Italy; 2 Fondazione Policlinico Universitario A. Gemelli IRCCS, 00168 Rome, Italy

**Keywords:** hippocampal adult neurogenesis, neural stem and progenitor cells, epigenetics, maternal HFD

## Abstract

Early-life metabolic stress has been demonstrated to affect brain development, persistently influence brain plasticity and to exert multigenerational effects on cognitive functions. However, the impact of an ancestor’s diet on the adult neurogenesis of their descendants has not yet been investigated. Here, we studied the effects of maternal high fat diet (HFD) on hippocampal adult neurogenesis and the proliferation of neural stem and progenitor cells (NSPCs) derived from the hippocampus of both the second and the third generations of progeny (F2_HFD_ and F3_HFD_). Maternal HFD caused a multigenerational depletion of neurogenic niche in F2_HFD_ and F3_HFD_ mice. Moreover, NSPCs derived from HFD descendants showed altered expression of genes regulating stem cell proliferation and neurodifferentiation (i.e., Hes1, NeuroD1, Bdnf). Finally, ancestor HFD-related hyper-activation of both STAT3 and STAT5 induced enhancement of their binding on the regulatory sequences of Gfap gene and an epigenetic switch from permissive to repressive chromatin on the promoter of the NeuroD1 gene. Collectively, our data indicate that maternal HFD multigenerationally affects hippocampal adult neurogenesis via an epigenetic derangement of pro-neurogenic gene expression in NSPCs.

## 1. Introduction

The brain is a high stress-sensitive organ, and it has now been recognized that early-life stress can affect brain plasticity in adult offspring [1]. Early nutritional experience together with stress hormones from the mother during the perinatal period may persistently regulate gene expression in the hippocampus via a plethora of mechanisms including epigenetic modifications [2]. Maternal overnutrition has been demonstrated to have lasting negative effects on both mature neurons and stem and progenitor cells (NSPCs) of the offspring, by inhibiting neurotrophic factor expression and promoting neuroinflammation [3,4,5]. We also reported that maternal high fat diet (HFD)-dependent insulin resistance caused multigenerational impairment of hippocampal synaptic plasticity and cognitive deficits resembling an Alzheimer’s disease-like phenotype until the third generation of descendants [6]. However, whether perinatal metabolic stress may multigenerationally impact on the proliferation of hippocampal NSPCs and the underlying molecular mechanisms remain largely unknown. NSPCs represent the cellular source of newborn neurons in the dentate gyrus of the hippocampus, and their fate is highly modulated by metabolic signals [7,8]. Epigenetic mechanisms are key factors controlling the neural fate of NSPCs and they dynamically regulate central nervous system development and adult neurogenesis [9,10]. Here, we demonstrate that maternal HFD multigenerationally impairs the hippocampal neurogenic niche in the descendants until the third generation. Moreover, the inhibition of NSPC proliferation appears to be regulated by cell-autonomous molecular mechanisms involving epigenetic inactivation of genes regulating proliferation and neurodifferentiation of NSPCs such as Hes1, NeuroD1 and Bdnf.

## 2. Materials and Methods

### 2.1. Animals

C57BL/6 mice (1 month old) were obtained from the Animal Facility of Università Cattolica del Sacro Cuore. Female mice (F0) were randomly assigned to two diet regimens: SD (control diet) and HFD and weighed weekly. F0 were paired for breeding at the end of the fourth week of dietary regimen, when HFD females showed metabolic alterations (i.e., increases of body weight, fasting glucose and insulin plasma levels) resembling a model of peripheral insulin resistance as previously described [6]. F0 pregnant females were fed with HFD until the second week of lactation. Male mice were always fed with standard chow. The same male mouse was paired, at different times, with both an F0 SD and an F0 HFD female mouse and was exposed to HFD only during this time lapse. A subset of male C57BL/6 offspring (F1_SD_ and F1_HFD_) was paired for breeding with control females giving rise to the second generation (F2_SD_ and F2_HFD_, respectively). Similarly, F2_SD_ and F2_HFD_ male mice were weaned onto standard chow and a subset of them was paired for breeding with control, SD-fed C57BL/6 females to produce the third generation (F3_SD_ and F3_HFD_). All SD descendants (F2 and F3) were indicated as SD or controls in figures and text. Different subgroups of male mice weaned onto standard chow were used for immunofluorescence analyses. For the preparation of NSPC cultures, different subsets of male and female pups were euthanized at 0–24 h after birth. A maximum of two male offspring was taken from each litter for each experimental set to remove any litter effects. Similar litters in term of number (6–8 pups) were used in our study to standardize competition for food and maternal care.

### 2.2. Diet and Housing Conditions

F0 C57BL/6 mice were fed with SD (18.5% proteins; 46% carbohydrates, namely 42% starch, 4% sucrose; 3% fats; 6.55% fat caloric content; cat. num. 4RF21) or HFD (23% proteins; 42% carbohydrates, namely 28% starch, 9% sucrose, 5% maltodextrin; 34% fats; 60% fat caloric content; cat. num. PF4051/D) for 4 weeks before mating, during pregnancy and until the second week of lactation (for a total of 9 weeks). Diets were from Mucedola (Settimo Milanese, MI, Italy). Diets were stored in refrigerators at 4 °C and chows in each cage were replaced every week to avoid deterioration. Mice were housed in 25 cm × 20 cm × 15 cm cages, under a 12-h light–dark cycle at room temperature (19–22 °C) and received water ad libitum. Weight and food consumption were weekly monitored.

### 2.3. NSPC Cultures 

NSPCs were isolated from hippocampal SGZ of male and female pups and cultured as described in previously published protocols [7]. Briefly, brains of 0–1 days old SD, F1_HFD_, F2_HFD_ and F3_HFD_ pups were microdissected and the hippocampal region was isolated through sagittal sectioning. Tissues were minced and incubated in a water bath at 37 °C for 30 min with accutase (in DPBS, 0.5 mM EDTA; Innovative Cell Technologies, Inc., San Diego, CA, USA) to ensure chemical digestion. After centrifugation at 800× *g* for 5 min, cells were mechanically dissociated and resuspended in Neurobasal-A medium supplemented with 2% B27 without vitamin A (Gibco), Glutamax (0.5 mM), recombinant mouse Fibroblast Growth Factor basic (10 ng/mL), recombinant human Epidermal Growth Factor (10 ng/mL), and recombinant mouse Platelet Derived Growth Factor-bb (10 ng/mL) (Thermo Fisher Scientific, Waltham, MA, USA). After resuspension, cells were seeded and placed in an incubator at 37 °C in a 5% CO_2_ atmosphere. Neurospheres began to form after one week of culture. Every 48 h, neurospheres were collected and enzymatically and mechanically dissociated to obtain monolayer cultures, which were gently plated onto Matrigel Matrix (Becton Dickinson, Franklin Lakes, NJ, USA) pre-coated Petri dishes. 

For neurosphere assay (NSA) experiments, undifferentiated proliferating NSPCs obtained by careful neurosphere dissociation were plated onto 96 well plated for 7 days before being analyzed with a phase contrast microscopy.

### 2.4. Real-Time PCR

RT-PCR experiments were performed using SYBR GREEN qPCR Master Mix (Fisher Molecular Biology, Rome, Italy) on AB7500 instrument (Life Technologies, Thermo Fisher Scientific, Waltham, MA, USA) according to the manufacturer’s instructions. The thermal cycling program consisted in a pre-incubation step of 94 °C for 10 min, followed by 40 cycles of denaturation (94 °C, 15 s), annealing (55 °C, 30 s), and elongation (72 °C, 20 s). For each RT-PCR experiment, only single products had been amplified, as confirmed by melting curves subsequently generated (94 °C for 15 s, 50 °C for 30 s, slow heating to 94 °C in increments of 0.5 °C). For PCR array experiments, the mRNA levels of 89 genes of interests plus 5 housekeeping genes were simultaneously examined through an RT2 Profiler Custom PCR Array (PAMM-126Z) in 96-well plates according to manufacturer’s protocol (Qiagen). Each experiment (one 96 well plate) included 40 ng of total extracted RNA and the negative controls (no template, no reverse transcription,). All samples were analyzed in triplicate, and data were normalized to glyceraldehyde 3-phosphate dehydrogenase levels using the ΔΔCt method. Results are shown in Supplementary Table S1.

### 2.5. Immunofluorescence Experiments

Animals were deeply anesthetized and transcardially perfused with 1X PBS (0.1 M, pH 7.4) and 4% PFA dissolved in ultra-pure H_2_O. Once collected, brains were first post-fixed for 16 h in 4% PFA at 4 °C in a refrigerator, and then incubated in a solution of 30% sucrose in 0.1 M PBS. Brain slices (35 μm thick) were cut coronally using a vibratome (VT1000S, Leica Microsystems, GmbH, Wetzlar, Germany) at RT. Regarding the BrdU/DCX double immunofluorescent labelling, sections were processed as described in previously published protocols [7]. Only Nestin immunolabeled slices were pre-incubated in 10 mM Sodium Citrate buffer pH 6.0 and 0.05% Tween at 60 °C in a water bath overnight, to perform an antigen retrieval step. Slices were then permeabilized and non-specific sites were blocked through an incubation step of 1 h in blocking buffer (1X PBS with 0.3% Triton X-100 (Sigma, St. Louis, MO, USA) and 5% NGS). After the blocking/permeabilization step, tissues were incubated overnight with gentle agitation at 4 °C with Nestin antibody (Abcam, 1:200) diluted in blocking buffer (1X PBS with 0.3% Triton X-100 and 5% NGS). The day after, tissues were incubated for 90 min at RT with the secondary antibody: Alexa Fluor-488 anti-mouse (1:500; Invitrogen, Carlsbad, CA, USA). Finally, nuclei were stained with DAPI (0.5 µg/mL for 10 min; Invitrogen, Carlsbad, CA, USA), and slices were coverslipped with ProLong Gold anti-fade reagent (Invitrogen). Antibodies are available in Supplementary Table S2.

### 2.6. Confocal Image Analysis

Images with a resolution of 1024 × 1024 pixels were acquired at a 20× magnification using a Nikon A1 MP confocal system (Tokyo, Japan) (Numerical Aperture 1.2). The relative scale bar is shown in every set of images.

For DCX^+^/BrdU^+^ immunofluorescence analyses, DAPI^+^/BrdU^+^ and DCX^+^/BrdU^+^ cells were counted. For analysis of Nestin immunolabeling, cells bodies showing an immunoreactivity for Nestin were counted and immunofluorescence intensity was calculated using ImageJ software following manufacturer’s instructions.

At least six hippocampal slices from each mouse brain were processed. *n* values indicate the number of studied mice.

### 2.7. Chromatin Immunoprecipitation

Plated NSPCs were washed in PBS 1X and resuspended in lysis buffer (1% sodium dodecyl sulfate (SDS), 50 mM Tris-HCl pH 8.0, and 10 mM EDTA). Samples were then sonicated on ice with the following protocol: six 10-s pulses with a 20-s interpulse interval. After a 20 min centrifugation, supernatants were incubated with protein-G Sepharose 4B beads (Sigma–Aldrich) for 1 h at 4 °C in a rotating wheel for the preclearing step. Two μg of primary antibody or control mouse/rabbit IgG were added and samples were incubated overnight at 4 °C. The day after, the protein/antibody complexes were incubated with protein-G Sepharose 4B beads for 2 h at 4 °C. After 5–7 sequential washes, protein/antibody complexes were eluted from beads by incubation in elution buffer (1% SDS and NaHCO3 0.1 M; pH 8.0) and vortexing. After an addition of 0.33M NaCl, cross-linking was reversed by an overnight incubation at 65 °C. DNA fragments were purified by using the PCR DNA Fragments Purification Kit (Geneaid). All primer sequences are shown in Supplementary Table S3. PCR conditions and cycle numbers were empirically determined, and each PCR reaction was performed in triplicate. Data are expressed as the percentage of input calculated by the adjusted input value method according to the manufacturer’s instructions (Thermo Fisher Scientific ChIP Analysis). To calculate the adjusted input, the Ct value of input was subtracted by 6.644 (i.e., log2 of 100). Next, the percentage of input of samples was calculated using the formula: 100 × 2^(Adjusted input − Ct(ChIP). The percentage of input of IgG samples was calculated using the formula 100 × 2^(Adjusted input—Ct(IgG).

### 2.8. Western Blotting

Plated NSPCs were homogenized in cold lysis buffer containing NaCl 150 mM, Tris-HCl 50mM pH 7.4, EDTA 2 mM and supplemented with 1% Triton X-100, 0.1% SDS, 1 × protease inhibitor cocktail (Sigma-Aldrich, Merck KGaA, Darmstadt, Germany), 1mM sodium orthovanadate (Sigma–Aldrich), and 1mM sodium fluoride (Sigma-Aldrich, Merck KGaA, Darmstadt, Germany). After a 10 min incubation on ice at 4 °C with occasional vortexing, homogenates were sonicated on ice for 5 min and centrifuged at 13,000× *g* for 20 min at 4 °C. Protein content in the supernatant was quantified through the Bradford assay (DC Protein Assay; Bio-Rad, Segrate, MI, Italy). Next, 40–60 μg of proteins from total lysates were diluted in Laemmli buffer, boiled for 5 min at 100 °C, and resolved using an SDS-PAGE polyacrylamide gel. The primary antibodies (1 μg/mL, diluted in TBS-Tween20, 3% non-fat dried milk) were incubated overnight at 4 °C on a plate shaker and revealed with horseradish peroxidase-conjugated secondary antibodies (1:5000 in TBS-Tween20, Cell Signaling Technology Inc., Danvers, MA, USA). Single protein expression was evaluated using UVItec Cambridge Alliance Software. All uncropped blots are included in Supplementary files. Antibodies are available in Supplementary Table S2.

### 2.9. Statistical Analysis

All statistical analyses, including sample size calculation, were performed using the software SigmaPlot 14.0. Sample sizes were estimated with adequate power (0.8) following results of prior pilot datasets or studies based on similar methods or paradigms, including our own. Prior to statistical tests, equal variance and normality (Shapiro–Wilk test) were assessed. The statistical tests used (i.e., Student’s *t*-test, one-way ANOVA) are reported in the main text and in the corresponding figure legends for each experiment. Post hoc multiple comparisons were performed with Bonferroni correction. The level of significance was set at 0.05 and all statistical tests were two-tailed. Results are expressed as mean ± SEM.

## 3. Results

### 3.1. Ancestor HFD Multigenerationally Impairs Hippocampal Adult Neurogenesis

**Figure 1:**
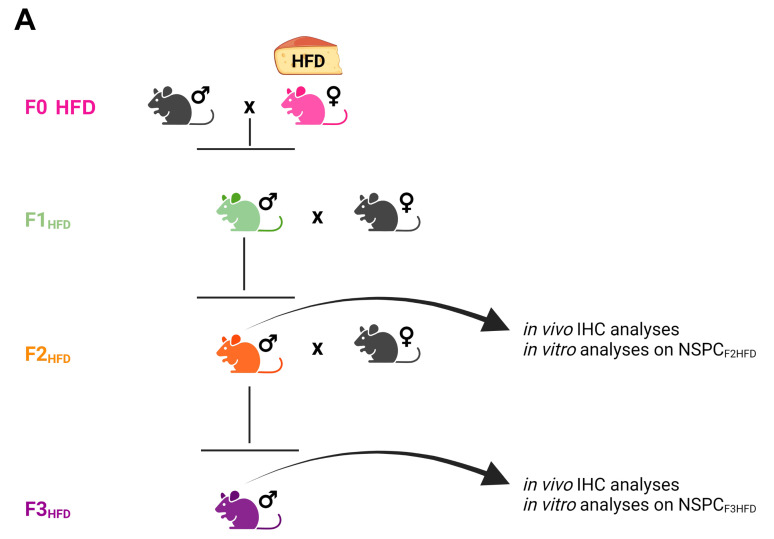
Experimental model. Female mice (F0) were fed with either standard diet (F0_SD_) or high-fat diet (F0_HFD_, pink) for 4 weeks before mating with control SD-fed male mice. HFD was maintained during the pregnancy and until the second week of lactation. The offspring (F1_HFD_, green) and descendants (F2_HFD_, orange and F3_HFD_, purple) were always fed with SD. F1_HFD_ and F2_HFD_ male mice were mated with SD-fed females to generate, respectively, F2_HFD_ and F3_HFD_ mice. All analyses were performed on F2_HFD_ and F3_HFD_ brains, and on NSPCs derived from their hippocampi (NSPC_F2HFD_ and NSPC_F3HFD_, respectively) and cultivated in vitro (see Materials and Methods section).

Maternal HFD during gestation and early stages of newborn life has been shown to alter the hippocampal plasticity of the offspring by impairing adult neurogenesis, dendritic spine formation, and memory [5,11,12]. We also demonstrated that HFD feeding during the early phase of life transgenerationally impaired hippocampal synaptic function until the third generation by epigenetically inhibiting the expression of BDNF in mature neurons. However, it is unknown whether the neurogenic niche in the subgranular zone (SGZ) of the dentate gyrus (DG) can also be multigenerationally affected by the dysmetabolic environment of the ancestor. To test this hypothesis, we fed female mice (F0) with standard diet (SD) or HFD for 4 weeks before mating, during the pregnancy, and until the second week of lactation. The offspring (F1_SD_ or F1_HFD_) were always fed with standard diet (SD) since the weaning. Male F1_SD_ and F1_HFD_ mice were bred with control females to produce a second filial generation (F2_SD_ and F2_HFD_, respectively), in which hippocampal adult neurogenesis was evaluated at the age of three months (Figure 1). 

**Figure 2:**
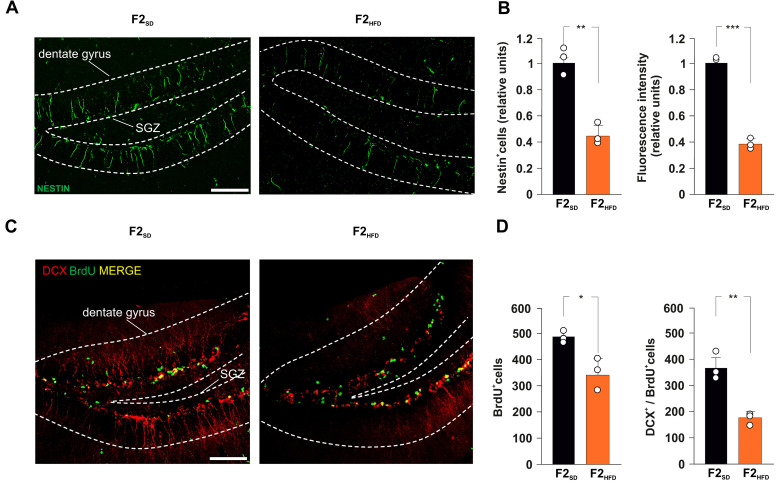
Ancestor HFD impairs adult hippocampal neurogenesis in F2_HFD_ descendants. (**A**) Representative images of Nestin^+^ cell immunostaining in hippocampal slices containing the subgranular zone (SGZ) of F2_SD_ and F2_HFD_ male mice (*n* = 3 mice per group; *n* = 6 slices per animal; statistics by unpaired Student’s *t* test). Scale bar = 50 μm. (**B**) Bar graphs showing the change of Nestin^+^ cell number (left) and Nestin fluorescence intensity (right) in F2_SD_ and F2_HFD_ mice. (**C**) Representative images of DCX^+^/BrdU^+^ cell immunostaining in hippocampal slices containing the SGZ of F2_SD_ and F2_HFD_ mice (*n* = 3 mice per group; *n* = 6 slices per animal; unpaired Student’s *t* test). Scale bar = 50 μm. (**D**) Bar graphs showing the number of BrdU^+^ cells (left) and the number of DCX^+^/BrdU^+^ cells (right) in F2_SD_ and F2_HFD_ mice (*n* = 3 mice per group; *n* = 6 slices per animal; statistics by unpaired Student’s *t* test). Data are expressed as mean ± SEM. * *p* < 0.05; ** *p* < 0.01; *** *p* < 0.001.

F2_HFD_ mice showed a severe decrease in the number of immunoreactive cells for the stemness-related protein Nestin in the DG (−55.3%, *p* = 0.0016; *n* = 3; Figure 2A,B). Moreover, we found a lower number of cells incorporating the proliferation marker BrdU in the SGZ of HFD descendants compared to controls (−30.1%, *p* = 0.0112; *n* = 3; Figure 2C,D). 

**Figure 3:**
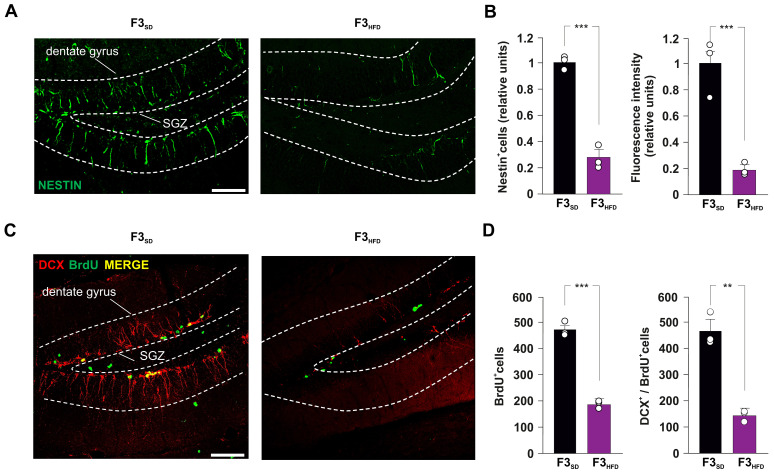
Ancestor HFD impairs adult hippocampal neurogenesis in F3_HFD_ descendants. (**A**) Representative images of Nestin^+^ cell immunostaining in hippocampal slices containing the SGZ of F3_SD_ and F3_HFD_ male mice (*n* = 3 mice per group; *n* = 6 slices per animal; statistics by unpaired Student’s *t*-test). Scale bar = 50 μm. (**B**) Bar graphs showing the change of Nestin^+^ cell number (left) and Nestin fluorescence intensity (right) in F3_SD_ and F3_HFD_ mice. (**C**) Representative images of DCX^+^/BrdU^+^ cell immunostaining in hippocampal slices containing SGZ of F3_SD_ and F3_HFD_ mice (*n* = 3 mice per group; *n* = 6 slices per animal; unpaired Student’s *t*-test). Scale bar = 50 μm. (**D**) Bar graphs showing the number of BrdU^+^ cells (left) and the number of DCX^+^/BrdU^+^ cells (right) in F3_SD_ and F3_HFD_ mice (n = 3 mice per group; *n* = 6 slices per animal; statistics by unpaired Student’s *t*-test). Data are expressed as mean ± SEM. ** *p* < 0.01; *** *p* < 0.001.

To evaluate the impact of ancestor’s metabolic stress on hippocampal adult neurogenesis, we also assessed the number of double-labeled cells for both BrdU and the marker of immature neurons doublecortin (DCX). Analysis of F2_HFD_ hippocampi revealed a significant decrease in BrdU^+^/DCX^+^ cells compared to F2_SD_ mice (−51.9%, *p* = 0.0043; *n* = 3; Figure 2D). Then, we continued to cross F2_SD_ and F2_HFD_ mice with control females to produce the third generation (F3_SD_ and F3_HFD_ mice, respectively), the first one that had no contacts with the dysmetabolic environment of the ancestor [13]. Strikingly, within the SGZ of DG derived from brain sections of F3_HFD_ mice we found alterations of both NSPC proliferation and differentiation similar to what observed in F2_HFD_ mice compared to SD animals (Nestin^+^ cells: −72.8%, *p* = 0.0006; BrdU^+^ cells: −60.1%, *p* = 5.53 × 10^−5^; BrdU^+^/DCX^+^ cells: −69.6%, *p* = 0.0011; *n* = 3; Figure 3A–D). Collectively, our ex vivo experiments indicated that metabolic stress occurring in the critical phase of brain development multigenerationally affected hippocampal adult neurogenesis.

### 3.2. Ancestor HFD Multigenerationally Alters Proliferation and Gene Expression in NSPCs

**Figure 4:**
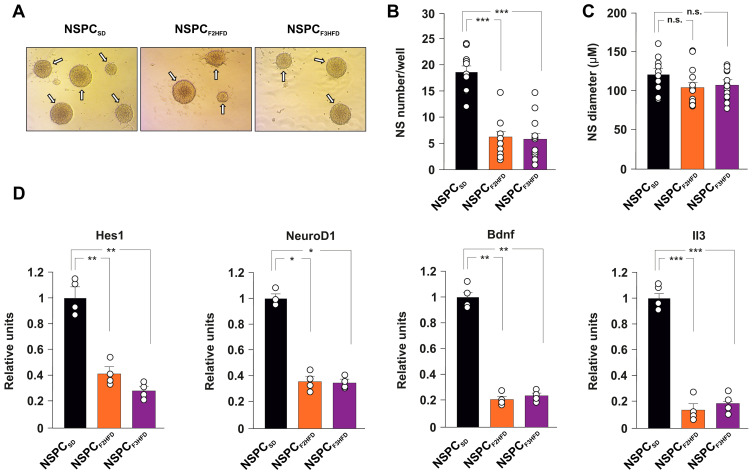
HFD descendants-derived NSPCs showed reduced proliferation and altered gene expression in vitro. (**A**) Bright-field images of SD, F2_HFD_ and F3_HFD_ mice derived-NSPCs (NSPC_SD_, NSPC_F2HFD_ and NSPC_F3HFD_, respectively). Scale bar = 100 μm. (**B**) Bar graphs showing the number and (**C**) mean diameter of neurospheres evaluated by neurosphere assay (statistics by one-way ANOVA and Bonferroni post hoc). (**D**) Bar graphs showing the expression levels of stemness-related genes significantly downregulated in both NSPC_F2HFD_ and NSPC_F3HFD_ (fold change ≥ 2 and *p*-value < 0.05; *n* = 4 mice per experimental group). Real-time (RT)-PCR was performed in triplicate. The full list of genes and fold expression changes are shown in Supplementary Table S1. Data are expressed as mean ± SEM. * *p* < 0.05; ** *p* < 0.01; *** *p* < 0.001; n.s. is not significant.

Neural stem cell fate is orchestrated by finely coordinated cell-intrinsic programs of gene expression and external signals within the neurogenic niche [14,15]. To dissect the role of cell autonomous and non-autonomous mechanisms underlying the inhibition of NSPC proliferation in HFD descendant brains, we set up in vitro cultures of NSPCs obtained from neonatal hippocampi isolated from SD and HFD descendant mice (hereinafter named NSPC_SD_, NSPC_F2HFD_ and NSPC_F3HFD_, Figure 1). We cultivated all NSPCs in the same medium and analyzed their proliferation by neurosphere assay (NSA, Figure 4A). Surprisingly, both NSPC_F2HFD_ and NSPC_F3HFD_ showed lower numbers of newformed neurospheres after one week compared to NSPC_SD_ (−66.6% and −68.8%, respectively, *p* < 0.001; *n* = 20; Figure 4B), indicating that even in vitro the NSPCs multigenerationally maintained the impairment of proliferation observed in the hippocampus of HFD descendants. No changes in the diameter of neurospheres were detected (Figure 4C). To gain insight into the molecular mechanisms underlying the ancestor HFD-dependent alteration of hippocampal adult neurogenesis, we investigated the expression of many NSPC fate-related genes in NSPC_SD_, NSPC_F2HFD_ and NSPC_F3HFD_. Real-time PCR array analysis showed either upregulation or downregulation of several genes in NSPCs obtained from all generations of HFD descendants (Supplementary Table S1). We found a significant decrease in the key genes driving NSPC proliferation and neurogenesis in both NSPC_F2HFD_ and NSPC_F3HFD_ (Hes1 −59% and −72%, NeuroD1 −64% and −65%, BDNF −79% and −76%, IL3 −86% and −81%, respectively; *n* = 3; Figure 4D). Collectively, our in vitro findings suggested that cell autonomous molecular changes occurring in the NSPCs derived from HFD descendants might be involved in the impairment of adult neurogenesis detected in their hippocampi.

### 3.3. HFD Multigenerationally Alters the Activation State of ERK, STAT3 and STAT5 Kinases 

**Figure 5:**
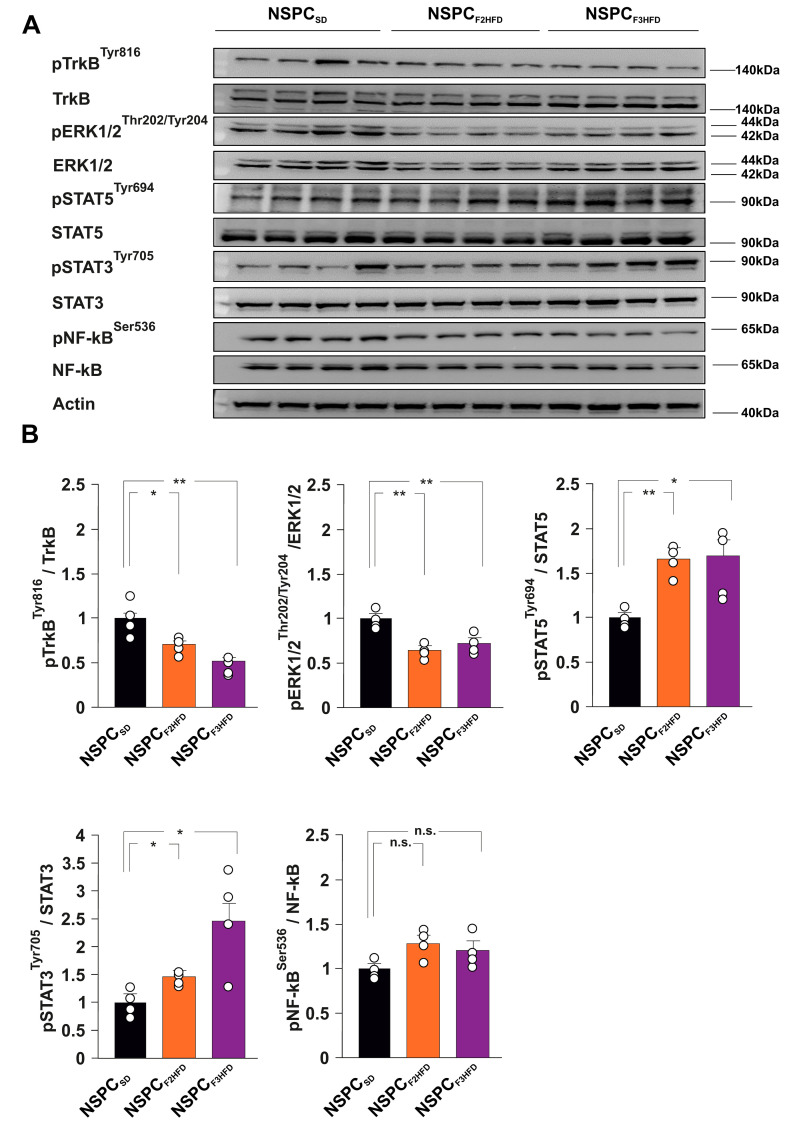
HFD descendants-derived NSPCs showed alterations of TrkB, ERK1/2, and STAT3/5 signaling. (**A**) Immunoblots and (**B**) bar graphs showing the levels of both expression and phosphorylation of TrkB, ERK1/2, STAT3, STAT5 and NF-κB (*n* = 4) in NSPC_SD_, NSPC_F2HFD_ and NSPC_F3HFD_ (statistics by one-way ANOVA and Bonferroni post hoc). Data are expressed as mean ± SEM. * *p* < 0.05; ** *p* < 0.01; n.s. is not significant.

Neurotrophic factors and interleukins represent some of the main growth factors regulating NSPC proliferation and differentiation [16,17]. Brain-derived neurotrophic factor (BDNF) has been demonstrated to foster the hippocampal neurogenic niche and to stimulate NSPC proliferation [18]. To understand the molecular cascades involved in the dysregulation of NSPC proliferation, we studied the activation state of several BDNF- and interleukin-downstream effectors in NSPC_SD_, NSPC_F2HFD_ and NSPC_F3HFD_. We found lower phosphorylation levels of both BDNF receptor TrkB and Extracellular signal-regulated kinases p44/p42 (ERK1/2) in NSPC_F2HFD_ and NSPC_F3HFD_ compared to controls (pTRKB^tyr816^: F_4.256_ = 13.458, −30.5% P = 0.0341 and −49%, *p* = 0.0034, respectively; pERK1/2^thr202/tyr204^: F_4.256_ = 15.584, −35% *p* = 0.0012 and −27%, *p* = 0.0087, respectively; *n* = 4; Figure 5A,B). 

Conversely, we observed enhanced activation of signal transducers and activators of transcription 3 and 5 (STAT3 and STAT5) in NSPCs derived from HFD descendants (pSTAT5^tyr694^: F_4.256_ = 9.101, +64%, *p* = 0.0012 in NSPC_F2HFD_ and +65%, *p* = 0.0163 in NSPC_F3HFD_; pSTAT3^tyr705^: F_4.256_ = 7.983, +44%, P = 0.0157 in NSPC_F2HFD_ and +146%, *p* = 0.0183 in NSPC_F3HFD_; *n* = 4; Figure 5A,B). No significant phosphorylation changes of inflammation-associated Nuclear Factor kappa B (NF-kB) were detected in NSPCs. Our data confirmed that maternal HFD multigenerationally induced alterations of intracellular molecular cascades involved in the regulation of NSPC behavior. 

### 3.4. Ancestor HFD Epigenetically Dysregulates the Promoters of Genes Driving Neurogenesis in Hippocampal NSPCs

**Figure 6:**
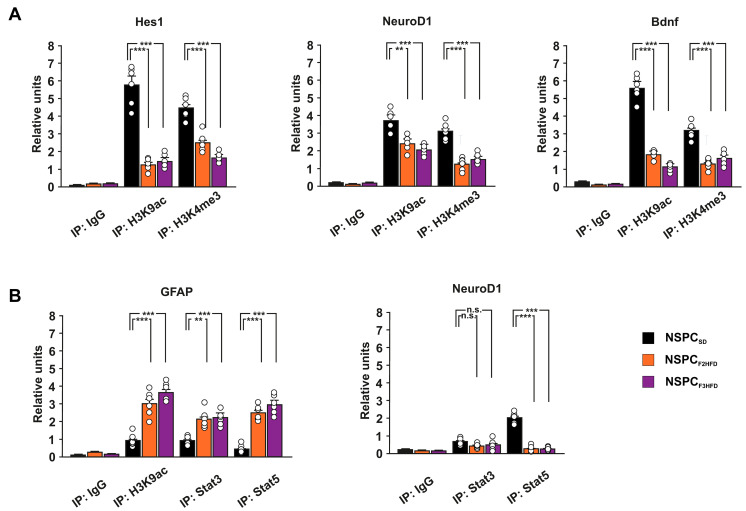
Ancestor HFD induced epigenetic alteration in the promoters of genes regulating adult neurogenesis. (**A**) ChIP assays of H3K9ac and H3K4me3 on the promoters of Hes1, NeuroD1, and Bdnf genes in NSPC_SD_, NSPC_F2HFD_ and NSPC_F3HFD_ (*n* = 6; statistics by one-way ANOVA and Bonferroni post hoc). (**B**) ChIP assays of H3K9ac, STAT3 and STAT5 on the promoter of Gfap, and STAT3/STAT5 on the promoter of NeuroD1 in NSPC_SD_, NSPC_F2HFD_ and NSPC_F3HFD_. (*n* = 6; statistics by one-way ANOVA and Bonferroni post hoc). IgG are used as negative controls. Real-time analysis was performed in triplicate. Data are expressed as mean ± SEM. ** *p* < 0.01; *** *p* < 0.001; n.s. is not significant.

Several soluble factors, including neurotrophic factors, can modulate adult neurogenesis and memory via autocrine/paracrine mechanisms [19,20,21]. We previously demonstrated that maternal HFD multigenerationally impaired cognitive functions by epigenetically inhibiting the expression of synaptic plasticity-related genes in the hippocampus of descendants until the third generation [6]. To investigate the role of epigenetic modifications in the HFD-dependent multigenerational inhibition of hippocampal neurogenesis, we analyzed the levels of transcriptional activity markers histone H3 lysine 9 acetylation (H3K9ac) and lysine 4 trimethylation (H3K4me3) on the regulatory sequences of Hes1, NeuroD1 and Bdnf genes. We found reduced levels of both H3K9ac and H3K4me3 on the promoters of the downregulated genes regulating proliferation and neurodifferentiation of NSPCs (Hes1 H3K9ac: F_3.682_ = 81.256, −78% in NSPC_F2HFD_, *p* = 6.79 × 10^−5^, −74% in NSPC_F3HFD_, *p* = 9.78 × 10^−5^; Hes1 H3K4me3: F_3.682_ = 52.387, −43.5% in NSPC_F2HFD_, *p* = 1.27 × 10^−4^, −62% in NSPC_F3HFD_, *p* = 1.66 × 10^−5^; NeuroD1 H3K9ac: F_3.682_ = 23.703, −35% in NSPC_F2HFD_, *p* = 0.0016, −45% in NSPC_F3HFD_, *p* = 3.27 × 10^−4^; NeuroD1 H3K4me3: F_3.682_ = 41.985, −60% in NSPC_F2HFD_, *p* = 2.84 × 10^−5^, −51% in NSPC_F3HFD_, *p* = 1.27 × 10^−4^; Bdnf H3K9ac: F_3.682_ = 189.814, −67% in NSPC_F2HFD_, *p* = 1.26 × 10^−5^, −80% in NSPC_F3HFD_, *p* = 4.52 × 10^−6^; Bdnf H3K4me3: F_3.682_ = 41.440, −58.5% in NSPC_F2HFD_, *p* = 6.51 × 10^−6^, −48.5% in NSPC_F3HFD_, *p* = 8.44 × 10^−5^; *n* = 6; Figure 6A). 

NSPCs derived from HFD descendants showed higher activation levels of transcription factors STAT3 and STAT5, which have been demonstrated to promote the differentiation of NSPC toward astrocytes [22]. Moreover, STAT3 has been proposed to differentially regulate the expression of glial versus neuron differentiating factors [23]. Thus, we analyzed the binding of both STAT3 and STAT5 on the promoters of Gfap and NeuroD1 genes. STAT3 and STAT5 enrichment occurred at the Gfap promoter in both NSPC_F2HFD_ and NSPC_F3HFD_ (STAT3: F_3.682_ = 18.663, NSPC_F2HFD_ vs. NSPC_SD_ *p* = 0.0012, NSPC_F3HFD_ vs. NSPC_SD_ *p* = 1.68 × 10^−4^; STAT5: F_3.682_ = 62.733, NSPC_F2HFD_ vs. NSPC_SD_ *p* = 5.29 × 10^−6^, NSPC_F3HFD_ vs. NSPC_SD_ *p* = 1.57 × 10^−5^; *n* = 6; Figure 6B). Accordingly, we found higher levels of transcriptional activity marker H3K9ac on the same regulatory sequence (F_3.682_ = 39.863, NSPC_F2HFD_ vs. NSPC_SD_ *p* = 2.09 × 10^−4^, NSPC_F3HFD_ vs. NSPC_SD_ *p* = 2.73 × 10^−6^; *n* = 6; Figure 6B). Conversely, we detected lower binding of the transcription factor STAT5 on the promoter of NeuroD1 gene in both NSPC_F2HFD_ and NSPC_F3HFD_ (F_3.682_ = 149.409, NSPC_F2HFD_ vs. NSPC_SD_ *p* = 4.33 × 10^−6^, NSPC_F3HFD_ vs. NSPC_SD_ *p* = 9.64 × 10^−6^; *n* = 6; Figure 6B). Thus, an epigenetic switch of transcription factor STAT recruitment on the promoters of NSPC differentiation genes may be involved in the ancestor HFD-dependent multigenerational inhibition of adult neurogenesis. Collectively, our results reveal that maternal HFD can multigenerationally impair hippocampal adult neurogenesis via cell-autonomous epigenetic modulation of genes regulating NSPC proliferation and differentiation.

## 4. Discussion

Pre- and perinatal exposure to different stressful conditions have been recognized as affecting central nervous system development, and influencing mood, cognitive decline, and brain health throughout adult life [24,25,26]. A lot of experimental evidence converges on a critical role for adult neurogenesis in stress response and behavior alteration in rodents [27,28]. Accordingly, early-life stress-mediated permanent inhibition of neurogenesis has been implicated in these functional deficits [29]. Maternal exposure to HFD induces alterations of neuroendocrine system leading to oxidative stress, neuroinflammation, and change in gut–brain axis functionality [30]. It has been shown that maternal overnutrition caused hippocampal dendritic remodeling and neurogenesis deficits in adult offspring [31,32]. However, whether HFD-related signals exert multigenerational effects on the hippocampal neurogenic niche remains largely unexplored. Here, we show that maternal HFD multigenerationally impairs NSC proliferation and adult neurogenesis in the hippocampus of second and third generations of descendants (named F2_HFD_ and F3_HFD_ mice). We used an experimental model inducing metabolic alterations resembling the human insulin resistance only in the HFD-fed ancestor female mice, while the SD-fed descendants did not show any metabolic change compared to controls [6]. Nonetheless, we found reduced levels of proliferating NSPCs and immature neurons in the SGZ of F2_HFD_ and F3_HFD_ hippocampi (Figure 2 and Figure 3). Non-genetic multigenerational inheritance of environment-induced phenotypic changes may involve different mechanisms including transmission of epigenetic modifications via the gametes, but also humoral factors such as hormones and cytokines, and even microbiota alterations, without the involvement of gametes [33,34]. 

It is still debated whether the mouse germline can retain and transmit across the generations several epigenetic changes such as histone modifications and DNA methylation or they are completely erased during embryo development [35,36,37]. We already noted that maternal HFD induced long-term cognitive deficits until the third generation of descendants via gametic epigenetic alterations [6]. However, we also found reduced levels of circulating BDNF in the plasma of HFD descendants, which might contribute to the impairment of brain plasticity. Moreover, other mechanisms including microbiota alteration and sperm non-coding RNAs have been proposed to play a critical role in the effects of maternal diet on brain functions of progeny [38,39]. To investigate whether cell-autonomous mechanisms were involved in the impairment of hippocampal adult neurogenesis of HFD progeny, we set up primary cultures of NSPCs derived from the hippocampi of newborn F2_HFD_ and F3_HFD_ mice (named NSPC_F2HFD_ and NSPC_F3HFD_, Figure 1). These in vitro experiments revealed lower proliferation and altered expression of genes regulating stemness and differentiation toward neuronal lineage in both NSPC_F2HFD_ and NSPC_F3HFD_ compared to controls (Figure 4). We focused our attention on Hes1, NeuroD1 and Bdnf genes, which we found inhibited in all HFD descendants and we previously demonstrated to be sensitive to nutrient-related signals [6,7]. Accordingly, the regulatory sequences of these genes showed reduced levels of H3K9ac and H3K4met3, which is an epigenetic landscape inhibiting gene expression (Figure 6A). 

Cellular adaptation to environmental stress involves a wide range of molecular mechanisms, including transcription. Epigenetic changes modulate the transcriptional response to stress, and in some cases, can preserve long-lasting memory of stress exposure, even up to the next generations [40]. Moreover, alteration of BDNF expression appeared to be part of a self-sustaining autocrine/paracrine mechanism as suggested by the downregulation of TrkB/ERK intracellular signaling (Figure 5A,B). Abnormal neurotrophin activity is a leading etiological hypothesis by which early-life adverse experiences persistently modify brain plasticity [41]. Therefore, early-life stress increased the gliogenesis in several brain areas including the hippocampus [42]. Accordingly, alteration of BDNF signaling and astroglial activation play a critical role in the diet-dependent dysregulation of neuroplasticity [43,44]. In NSPC_F2HFD_ and NSPC_F3HFD_, we also found hyperactivation of STAT3 and STAT5, which have been reported to be triggered by HFD and promote the differentiation of NSPCs toward the astrocyte lineage via epigenetic mechanisms [22,45,46], suggesting a potential role of these transcription factors in the impairment of the adult neurogenesis we observed in the hippocampus of HFD descendants. ChIP assay revealed a molecular shift of STAT proteins occurring between NeuroD1 and Gfap promoters, in parallel with both epigenetic repression of pro-neurogenic and activation of pro-glial differentiation genes (Figure 6B). This epigenetic rearrangement of STAT transcription factors on the regulatory sequences of genes regulating NSPC commitment could be a tentative but ineffective metabolic stress response aimed at fostering and expanding the neurogenic niche [42]. Moreover, the inhibition of BDNF signaling together with alteration of redox state or cytokine-related signals may lead to the STAT reassembly with chromatin remodeling complexes triggering astrocyte-specific gene expression in NSPCs [47,48]. Collectively, our data indicate that ancestor HFD multigenerationally impairs adult neurogenesis and the epigenetic alterations occurring in hippocampal NSPCs may contribute to the derangement of the neurogenic niche.

NSPC proliferation, as well as adult neurogenesis, are controlled by extrinsic and intrinsic factors, and the understanding of their underlying mechanisms, which have not been completely elucidated, may offer novel insights for regenerative medicine [49]. Early-life stressful experiences affect adult neurogenesis and impact on the neuro-immuno-endocrine system, leading to an increased vulnerability to developing mood disorders and age-related cognitive decline [29]. Further studies aimed at identifying the epigenetic changes inherited over generations and the molecular mechanisms involved in the transgenerational transmission of brain vulnerability will be necessary to increase our knowledge about heritable traits mediating the susceptibility to brain diseases such as neurodegenerative disorders.

## Data Availability

The data presented in this study are available in Supplementary Table S1.

## Supplementary Materials

The following supporting information can be downloaded at: https://www.mdpi.com/article/10.3390/cells11172661/s1, Table S1: Gene regulation; Table S2: Antibodies; Table S3: Primers used for chip analyses.
