# Supplementary material for: High Fat Diet Multigenerationally Affects Hippocampal Neural Stem Cell Proliferation via Epigenetic Mechanisms
Source: Cells. 2022 Aug 27;11(17):2661. doi: 10.3390/cells11172661 (PMC9454549; doi:10.3390/cells11172661)
Supplement: Supplementary file 1 [file cells-11-02661-s001.zip › cells-1871643-supplementary.pdf]

Supplementary Table S1. GENE REGULATION

Refseq	Gene Symbol	Description	Fold Regulation F2	Fold Regulation F3
NM_009599	Ache	Acetylcholinesterase	1.69	1.09
NM_001008533	Adora1	Adenosine A1 receptor	1.15	-1.01
NM_009630	Adora2a	Adenosine A2a receptor	1.08	**-3.43**
NM_007439	Alk	Anaplastic lymphoma kinase	1.44	**-2.61**
NM_009685	Apbb1	Amyloid beta (A4) precursor protein-binding, family B, member 1	**2.99**	1.18
NM_009696	Apoe	Apolipoprotein E	**5.61**	**3.81**
NM_007471	App	Amyloid beta (A4) precursor protein	1.50	1.05
NM_009711	Artn	Artemin	-1.30	-1.09
NM_008553	Ascl1	Achaete-scute complex homolog 1 (Drosophila)	-1.26	-1.55
NM_009741	Bcl2	B-cell leukemia/lymphoma 2	1.55	-1.08
NM_007540	Bdnf	Brain derived neurotrophic factor	**-4.80**	**-4.13**
NM_007553	Bmp2	Bone morphogenetic protein 2	1.96	**2.07**
NM_007554	Bmp4	Bone morphogenetic protein 4	**2.60**	**5.91**
NM_007559	Bmp8b	Bone morphogenetic protein 8b	-1.02	-1.34
NM_009871	Cdk5r1	Cyclin-dependent kinase 5, regulatory subunit 1 (p35)	-1.04	-1.41
NM_145990	Cdk5rap2	CDK5 regulatory subunit associated protein 2	-1.03	-1.62
NM_203491	Chrm2	Cholinergic receptor, muscarinic 2, cardiac	-1.45	**-2.87**
NM_133828	Creb1	CAMP responsive element binding protein 1	-1.17	-1.56
NM_008176	Cxcl1	Chemokine (C-X-C motif) ligand 1	**2.84**	**4.43**
NM_010025	Dcx	Doublecortin	-1.45	-1.72
NM_007864	Dlg4	Discs, large homolog 4 (Drosophila)	1.51	-1.69
NM_007865	Dll1	Delta-like 1 (Drosophila)	**3.73**	-1.49
NM_010077	Drd2	Dopamine receptor D2	-1.74	**-6.08**
NM_007889	Dvl3	Dishevelled 3, dsh homolog (Drosophila)	1.38	1.29
NM_010110	Efnb1	Ephrin B1	**2.80**	1.39
NM_010113	Egf	Epidermal growth factor	1.04	-1.40
NM_177821	Ep300	E1A binding protein p300	1.06	-1.28
NM_001003817	Erbb2	V-erb-b2 erythroblastic leukemia viral oncogene homolog 2, neuro/glioblastoma derived oncogene homolog (avian)	1.37	-1.16
NM_008006	Fgf2	Fibroblast growth factor 2	-1.24	1.28
NM_010227	Flna	Filamin, alpha	1.34	-1.27
NM_010275	Gdnf	Glial cell line derived neurotrophic factor	1.63	**2.09**
NM_008155	Gpi1	Glucose phosphate isomerase 1	1.68	-1.02
NM_008169	Grin1	Glutamate receptor, ionotropic, NMDA1 (zeta 1)	-1.42	-1.43
NM_207225	Hdac4	Histone deacetylase 4	1.40	1.13
NM_008235	Hes1	Hairy and enhancer of split 1 (Drosophila)	**-2.45**	**-3.56**
NM_010423	Hey1	Hairy/enhancer-of-split related with YRPW motif 1	**2.19**	1.11

NM_013904	Hey2	Hairy/enhancer-of-split related with YRPW motif 2	1.55	-1.44
NM_013905	Heyl	Hairy/enhancer-of-split related with YRPW motif-like	**-2.68**	1.43
NM_010556	Il3	Interleukin 3	**-6.93**	**-5.31**
NM_010784	Mdk	Midkine	1.52	-1.23
NM_025282	Mef2c	Myocyte enhancer factor 2C	1.08	**-2.16**
NM_001081049	Kmt2a	Myeloid/lymphoid or mixed-lineage leukemia 1	1.51	-1.37
NM_001039934	Map2	Microtubule-associated protein 2	-1.37	-1.85
NM_010882	Ndn	Necdin	1.45	-1.03
NM_010883	Ndp	Norrie disease (pseudoglioma) (human)	1.41	1.28
NM_010894	Neurod1	Neurogenic differentiation 1	**-2.74**	**-2.84**
NM_010896	Neurog1	Neurogenin 1	1.79	-1.14
NM_009718	Neurog2	Neurogenin 2	**4.14**	1.44
NM_010897	Nf1	Neurofibromatosis 1	1.10	-1.31
NM_008711	Nog	Noggin	1.59	-1.15
NM_008714	Notch1	Notch gene homolog 1 (Drosophila)	1.58	-1.02
NM_010928	Notch2	Notch gene homolog 2 (Drosophila)	1.88	-1.25
NM_013708	Nr2e3	Nuclear receptor subfamily 2, group E, member 3	1.76	1.27
NM_176930	Nrcam	Neuron-glia-CAM-related cell adhesion molecule	-1.29	-1.63
NM_178591	Nrg1	Neuregulin 1	1.31	-1.23
NM_008737	Nrp1	Neuropilin 1	-1.68	-1.59
NM_010939	Nrp2	Neuropilin 2	1.17	-1.12
NM_008742	Ntf3	Neurotrophin 3	**-3.24**	**-2.04**
NM_008744	Ntn1	Netrin 1	1.09	-1.73
NM_011855	Tenm1	Odd Oz/ten-m homolog 1 (Drosophila)	**-2.38**	**-2.08**
NM_016967	Olig2	Oligodendrocyte transcription factor 2	1.03	-1.28
NM_013625	Pafah1b1	Platelet-activating factor acetylhydrolase, isoform 1b, subunit 1	-1.48	-1.53
NM_033620	Pard3	Par-3 (partitioning defective 3) homolog (C. elegans)	1.29	-1.00
NM_008781	Pax3	Paired box gene 3	**2.90**	**2.49**
NM_008782	Pax5	Paired box gene 5	1.10	1.80
NM_013627	Pax6	Paired box gene 6	1.33	-1.49
NM_008900	Pou3f3	POU domain, class 3, transcription factor 3	1.43	-1.20
NM_011143	Pou4f1	POU domain, class 4, transcription factor 1	1.01	-1.05
NM_008973	Ptn	Pleiotrophin	-1.10	-1.53
NM_009007	Rac1	RAS-related C3 botulinum substrate 1	-1.31	-1.49
NM_019413	Robo1	Roundabout homolog 1 (Drosophila)	-1.52	**-2.32**
NM_194053	Rtn4	Reticulon 4	1.23	1.10
NM_011313	S100a6	S100 calcium binding protein A6 (calcyclin)	1.38	-1.26
NM_009115	S100b	S100 protein, beta polypeptide, neural	-1.32	1.04
NM_009170	Shh	Sonic hedgehog	-1.25	-1.11
NM_178804	Slit2	Slit homolog 2 (Drosophila)	1.64	-1.17
NM_011434	Sod1	Superoxide dismutase 1, soluble	-1.40	-1.69
NM_011443	Sox2	SRY-box containing gene 2	**2.29**	1.16
NM_009237	Sox3	SRY-box containing gene 3	-1.07	-1.46

NM_011486	Stat3	Signal transducer and activator of transcription 3	1.33	-1.13
NM_011577	Tgfb1	Transforming growth factor, beta 1	1.63	**2.70**
NM_009377	Th	Tyrosine hydroxylase	**2.55**	**5.42**
NM_022312	Tnr	Tenascin R	**2.44**	**2.96**
NM_009505	Vegfa	Vascular endothelial growth factor A	1.11	-1.26

Refseq	Gene Symbol	Description	p-Value F2	p-Value F3
NM_009599	Ache	Acetylcholinesterase	**0.010453**	0.594951
NM_001008533	Adora1	Adenosine A1 receptor	0.609714	0.829176
NM_009630	Adora2a	Adenosine A2a receptor	0.593157	**0.007375**
NM_007439	Alk	Anaplastic lymphoma kinase	0.101612	**0.002003**
NM_009685	Apbb1	Amyloid beta (A4) precursor protein-binding, family B, member 1	**0.000057**	0.470665
NM_009696	Apoe	Apolipoprotein E	**0.000077**	**0.017986**
NM_007471	App	Amyloid beta (A4) precursor protein	**0.003269**	0.688932
NM_009711	Artn	Artemin	0.210884	0.870425
NM_008553	Ascl1	Achaete-scute complex homolog 1 (Drosophila)	0.187052	0.061668
NM_009741	Bcl2	B-cell leukemia/lymphoma 2	0.240524	0.903190
NM_007540	Bdnf	Brain derived neurotrophic factor	**0.008586**	**0.009831**
NM_007553	Bmp2	Bone morphogenetic protein 2	**0.028418**	0.183004
NM_007554	Bmp4	Bone morphogenetic protein 4	**0.009845**	**0.000982**
NM_007559	Bmp8b	Bone morphogenetic protein 8b	0.837673	0.424797
NM_009871	Cdk5r1	Cyclin-dependent kinase 5, regulatory subunit 1 (p35)	0.504518	0.133792
NM_145990	Cdk5rap2	CDK5 regulatory subunit associated protein 2	0.878202	0.088653
NM_203491	Chrm2	Cholinergic receptor, muscarinic 2, cardiac	0.092246	**0.030356**
NM_133828	Creb1	CAMP responsive element binding protein 1	0.061695	**0.032563**
NM_008176	Cxcl1	Chemokine (C-X-C motif) ligand 1	**0.030797**	**0.018344**
NM_010025	Dcx	Doublecortin	0.067358	0.342946
NM_007864	Dlg4	Discs, large homolog 4 (Drosophila)	**0.002828**	0.098017
NM_007865	Dll1	Delta-like 1 (Drosophila)	**0.000877**	0.259892
NM_010077	Drd2	Dopamine receptor D2	0.205205	0.161424
NM_007889	Dvl3	Dishevelled 3, dsh homolog (Drosophila)	**0.043163**	0.141284
NM_010110	Efnb1	Ephrin B1	**0.038293**	0.833732
NM_010113	Egf	Epidermal growth factor	0.846458	0.715483
NM_177821	Ep300	E1A binding protein p300	0.447920	0.233575
NM_001003817	Erbb2	V-erb-b2 erythroblastic leukemia viral oncogene homolog 2, neuro/glioblastoma derived oncogene homolog (avian)	**0.025476**	0.619125
NM_008006	Fgf2	Fibroblast growth factor 2	0.230545	0.391488
NM_010227	Flna	Filamin, alpha	**0.014368**	0.579934
NM_010275	Gdnf	Glial cell line derived neurotrophic factor	0.205894	**0.046243**
NM_008155	Gpi1	Glucose phosphate isomerase 1	**0.000654**	0.990410
NM_008169	Grin1	Glutamate receptor, ionotropic, NMDA1 (zeta 1)	0.055200	0.660069
NM_207225	Hdac4	Histone deacetylase 4	0.114299	0.590060
NM_008235	Hes1	Hairy and enhancer of split 1 (Drosophila)	**0.002417**	**0.004578**

NM_010423	Hey1	Hairy/enhancer-of-split related with YRPW motif 1	**0.000131**	0.559948
NM_013904	Hey2	Hairy/enhancer-of-split related with YRPW motif 2	**0.026353**	0.311251
NM_013905	Heyl	Hairy/enhancer-of-split related with YRPW motif-like	**0.034747**	0.265319
NM_010556	Il3	Interleukin 3	**0.000380**	**0.000750**
NM_010784	Mdk	Midkine	**0.042699**	0.258520
NM_025282	Mef2c	Myocyte enhancer factor 2C	0.976732	0.432114
NM_001081049	Kmt2a	Myeloid/lymphoid or mixed-lineage leukemia 1	**0.009046**	0.221820
NM_001039934	Map2	Microtubule-associated protein 2	**0.005279**	**0.002603**
NM_010882	Ndn	Necdin	0.065498	0.964958
NM_010883	Ndp	Norrie disease (pseudoglioma) (human)	**0.015369**	0.376061
NM_010894	Neurod1	Neurogenic differentiation 1	**0.026302**	**0.040129**
NM_010896	Neurog1	Neurogenin 1	0.325525	0.585378
NM_009718	Neurog2	Neurogenin 2	**0.002448**	0.396913
NM_010897	Nf1	Neurofibromatosis 1	0.483597	0.140604
NM_008711	Nog	Noggin	0.226445	0.618572
NM_008714	Notch1	Notch gene homolog 1 (Drosophila)	**0.000195**	0.880209
NM_010928	Notch2	Notch gene homolog 2 (Drosophila)	**0.000446**	0.414469
NM_013708	Nr2e3	Nuclear receptor subfamily 2, group E, member 3	0.368136	0.626982
NM_176930	Nrcam	Neuron-glia-CAM-related cell adhesion molecule	0.286506	0.112798
NM_178591	Nrg1	Neuregulin 1	0.221009	0.332660
NM_008737	Nrp1	Neuropilin 1	0.148567	0.215944
NM_010939	Nrp2	Neuropilin 2	0.386646	0.819174
NM_008742	Ntf3	Neurotrophin 3	**0.002056**	0.811532
NM_008744	Ntn1	Netrin 1	0.073181	0.202567
NM_011855	Tenm1	Odd Oz/ten-m homolog 1 (Drosophila)	**0.000780**	0.896029
NM_016967	Olig2	Oligodendrocyte transcription factor 2	0.870439	0.614556
NM_013625	Pafah1b1	Platelet-activating factor acetylhydrolase, isoform 1b, subunit 1	**0.043168**	**0.028524**
NM_033620	Pard3	Par-3 (partitioning defective 3) homolog (C. elegans)	**0.012435**	0.878701
NM_008781	Pax3	Paired box gene 3	0.321386	0.358581
NM_008782	Pax5	Paired box gene 5	0.611445	0.074594
NM_013627	Pax6	Paired box gene 6	0.109452	0.113203
NM_008900	Pou3f3	POU domain, class 3, transcription factor 3	**0.031213**	0.377303
NM_011143	Pou4f1	POU domain, class 4, transcription factor 1	0.800199	0.627075
NM_008973	Ptn	Pleiotrophin	0.312049	**0.007995**
NM_009007	Rac1	RAS-related C3 botulinum substrate 1	0.053404	**0.039678**
NM_019413	Robo1	Roundabout homolog 1 (Drosophila)	0.136546	**0.042143**
NM_194053	Rtn4	Reticulon 4	**0.010608**	0.560469
NM_011313	S100a6	S100 calcium binding protein A6 (calcyclin)	0.411202	0.367269
NM_009115	S100b	S100 protein, beta polypeptide, neural	0.359924	0.974769
NM_009170	Shh	Sonic hedgehog	0.429492	0.746186
NM_178804	Slit2	Slit homolog 2 (Drosophila)	**0.002514**	0.962489
NM_011434	Sod1	Superoxide dismutase 1, soluble	0.117487	**0.044359**
NM_011443	Sox2	SRY-box containing gene 2	**0.000052**	0.281332

NM_009237	Sox3	SRY-box containing gene 3	0.461867	0.217372
NM_011486	Stat3	Signal transducer and activator of transcription 3	**0.002053**	0.713467
NM_011577	Tgfb1	Transforming growth factor, beta 1	**0.005999**	**0.006445**
NM_009377	Th	Tyrosine hydroxylase	0.383059	0.072158
NM_022312	Tnr	Tenascin R	**0.000804**	**0.034151**
NM_009505	Vegfa	Vascular endothelial growth factor A	0.262642	0.224088

Supplementary Table S2. ANTIBODIES

Primary Antibody	Host	Catalogue Reference
Nestin (RAT 401)	Mouse	ABCAM ab11306
DCX	Rabbit	Cell Signaling Technology #4604S
BrdU	Rat	ABCAM ab6326
pERK1/2$^{Thr202/Tyr204}$	Rabbit	Cell Signaling Technology #9101
ERK1/2	Rabbit	Cell Signaling Technology #9102
pTrkB^{Tyr816}	Rabbit	Antibody against phospho-TrkB Tyr816 was a kind gift of Moses V. Chao
TrkB (80E3)	Rabbit	Cell Signaling Technology #4603
Actin	Rabbit	ABCAM ab8227
pSTAT5^{Tyr694}	Mouse	Cell Signaling Technology #9356
STAT5	Rabbit	Cell Signaling Technology #94205
pSTAT3^{Tyr705}	Rabbit	Cell Signaling Technology #9145
STAT3	Rabbit	Cell Signaling Technology #9139
pNF-kB^{Ser536} Clone:EP2294Y	Rabbit	ABCAM ab76302
NF-kB p65	Rabbit	ABCAM ab16502

Supplementary Table S3. PRIMERS USED FOR CHIP ANALYSES

Gene	Primer Sequences
GFAP	**FW** CAGGCCTTGTCTGTAAGCTGAAGAC
	**REV** TTATCCCAGGATGCCAGGATGTCAG
HES1	**FW** AAGTAGTTATATTGCATGCAGC
	**REV** AGATCCTGTGTGATCCGCAG
NEUROD1	**FW** TGCCCACAAG TACTCGCACG
	**REV** AGAACTGGAAGTGGTTGTTTGC
BDNF	**FW** GAAGCTGAGCTCTCTGTGG
	**REV** GGTCTCATGAAGAAACTTAAGG
